# Efficacy of carvacrol against resistant rapidly growing mycobacteria in the planktonic and biofilm growth mode

**DOI:** 10.1371/journal.pone.0219038

**Published:** 2019-07-01

**Authors:** Emanuela Marini, Mara Di Giulio, Giovanna Ginestra, Gloria Magi, Silvia Di Lodovico, Andreana Marino, Bruna Facinelli, Luigina Cellini, Antonia Nostro

**Affiliations:** 1 Unit of Microbiology, Department of Biomedical Sciences and Public Health, Polytechnic University of Marche, Ancona, Italy; 2 Department of Pharmacy, University "G. d'Annunzio" Chieti-Pescara, Chieti, Italy; 3 Department of Chemical, Biological, Pharmaceutical and Environmental Sciences, University of Messina, Messina, Italy; University of Hong Kong, HONG KONG

## Abstract

Rapidly growing mycobacteria (RGM) are environmental bacteria found worldwide with a propensity to produce skin and soft-tissue infections. Among them, the most clinically relevant species is *Mycobacterium abscessus*. Multiple resistance to antibiotics and the ability to form biofilm contributes considerably to the treatment failure. The search of novel anti-mycobacterial agents for the control of biofilm growth mode is crucial. The aim of the present study was to evaluate the activity of carvacrol (CAR) against planktonic and biofilm cells of resistant RGM strains. The susceptibility of RGM strains (n = 11) to antibiotics and CAR was assessed by MIC/MBC evaluation. The CAR activity was estimated by also vapour contact assay. The effect on biofilm formation and preformed biofilm was measured by evaluation of bacterial growth, biofilm biomass and biofilm metabolic activity. MIC values were equal to 64 μg/mL for most of RGM isolates (32–512 μg/mL), MBCs were 2–4 times higher than MICs, and MICs of vapours were lower (16 μg/mL for most RGM isolates) than MICs in liquid phase. Regarding the biofilm, CAR at concentrations of 1/2 × MIC and 1/4 × MIC showed a strong inhibition of biofilm formation (61–77%) and at concentration above the MIC (2–8 × MIC) produced significant inhibition of 4- and 8-day preformed biofilms. In conclusion, CAR could have a potential use, also in vapour phase, for the control of RGM.

## Introduction

Rapidly growing mycobacteria (RGM) are environmental bacteria capable of causing a wide spectrum of infections [1]. Among them, *Mycobacterium abscessus* is an emerging human pathogen causing lung infection but also responsible for wound, catheter and eye infections and also tattooing [2]. *Mycobacterium chelonae* is commonly associated with skin and soft tissue infections and also causes catheter-related and post-surgical infections; invasive infections are common in immunosuppressed patients but pulmonary infections are rare when compared to *M*. *abscessus* [3]. *Mycobacterium fortuitum* is responsible for the most of post-surgical wound and catheter infections produced by the RGM [4]. *Mycobacterium smegmatis*, previously considered an environmental saprophyte without any clinical significance, is accountable for community-acquired disease including cellulitis, localized abscesses and osteomyelitis [5]. *Mycobacterium mucogenicum* causes osteomyelitis and respiratory tract, bloodstream and disseminated infections in both immunocompetent and immunocompromised hosts [4].

Most of the *M*. *chelonae*, *M*. *abscessus* and *M*. *mucogenicum* isolates are widely resistant to antibiotics and disinfectants [6,7]. In particular, *M*. *abscessus* is resistant to the common and currently existing antibiotics [5]. An important virulence factor for RGM is the production of biofilms, both in medical and in environmental settings [8], that contribute to the therapy failure and relapses [9]. Notably, smooth *M*. *abscessus* strains are major biofilm-producers respect to rough *M*. *abscessus* strains [10].

The volatile monoterpene carvacrol (CAR) [2-Methyl-5-(1-methylethyl) phenol], a major constituent of many essential oils of the Labiatae family, is classified among the substances generally recognized as safe (GRAS) and approved for use in food [11]. Several studies have demonstrated its biological properties such as antioxidant, anti-inflammatory, antitumor, analgesic, anti-hepatotoxic and insecticidal activities [12–15]. Carvacrol has been known for its wide antimicrobial activity against food or pathogenic microorganisms, including drug-resistant bacteria [8,16,17]. CAR is also efficacious against organisms in the biofilm growth mode [18]. Specifically, it is able to interfere with biofilm growth of clinically relevant *Staphylococcus aureus* and *S*. *epidermidis* [19–21], *Salmonella tiphymurium* [22], *Listeria monocytogenes* [23] titanium-adherent oral microrganisms [24] and carbapenemase-producing Gram negative bacilli. The efficacy of CAR against microbial fungal biofilms has also been investigated [25]. Despite extensive research on the monoterpenic phenol CAR, there is little information on its efficacy against RGM. To our knowledge, the activity of CAR has been documented against fast-growing *M*. *phlei* ATCC 11758, *M*. *smegmatis* ATCC 19420 and *M*. *fortuitum* ATCC 6841 (MICs of 80–100 μg/mL) [26].

The goal of the present study was to extend the research and to evaluate the antimicrobial activity of CAR, either in liquid and vapour phase, against different species of resistant RGM. Moreover, the antibiofilm activity of CAR, in liquid phase, was also evaluated against RGM species capable to form biofilm.

## Materials and methods

### RGM strains and growth media

Eleven anonymized clinical strains of RGM were used for this study. Strains, were stored in the private collection of Unit of Microbiology, Department of Biomedical Sciences and Public Health, Polytechnic University of Marche, Ancona, Italy. Rapidly growing mycobacteria strains were identified as smooth morphotypes *M*. *abscessus* #09716, #29904, #30235, #70513, #73596, #90459, #74600; *M*. *chelonae* #74471; *M*. *fortuitum* #26647; *M*. *mucogenicum* #45646 and *M*. *smegmatis* #44041 by line-probe reverse hybridization assay (GenoType CM, Hain Lifescience, Nehren, Germany) and conventional biochemical and cultural methods, as suggested by Clinical and Laboratory Standard Institute (CLSI) [27]. Blood agar base (BAB, Oxoid, Basingstoke, UK) and Müller-Hinton cation-adjusted agar (CAMHA, Oxoid), both supplemented with 5% sheep blood; Müller-Hinton cation-adjusted broth (CAMHB, Oxoid); Middlebrook broth (MBB, Oxoid) and agar (MBA, Oxoid), both supplemented with 10% oleic albumin dextrose catalase (OADC, Oxoid) and 0.5% glycerol, were used for the experiments. Bacterial isolates were stored in cryovials with glycerol (20% v/v glycerol).

### Susceptibility of RGM strains to antibiotics and CAR

All antibiotics: amikacin (AMK), cefoxitin (FOX), ciprofloxacin (CIP), clarithromycin (CLR), linezolid (LZD), meropenem (MEM), sulfamethoxazole (SX) and tigecycline (TGC) as well as carvacrol (CAR; W224502, purity ≥98%) were purchased from Sigma-Aldrich (St. Louis, MO, USA). Antibiotics stock solutions (10 mg/mL) were stored in absolute ethanol at -20°C. Doubling broth dilutions of antibiotics and CAR were prepared in 96-well microtitre plates. In order to categorize RGM strains as susceptible, intermediate or resistant, antibiotic concentrations two-fold dilutions away from their breakpoints (Table 1) [28] were used in susceptibility testing. Currently there are no interpretative breakpoints available for meropenem, ertapenem and doripenem antibiotics for which those related to imipenem are considered [29]. Breakpoints for tigecycline were based on those used by Ananta [30].

**Table 1:** Antibiotic breakpoints used for interpretation of RGM susceptibility.

Antibiotics	MIC (μg/ml)		
	Susceptible	Intermediate	Resistant
AMK	≤16	32	≥64
FOX	≤16	32–64	≥128
CIP	≤1	2	≥4
CLR	≤2	4	≥8
LZD	≤8	16	≥32
MEM	≤4	8	≥16
SX	≤32	-	≥64
TGC	≤1	2	≥4

The final concentration of CAR ranging from 4096 to 8 μg/mL. Ethanol maximum concentration was 1.25% (v/v). Minimum inhibitory concentration (MIC) was determined by microdilution methods and interpreted following the Clinical and Laboratory Standards Institute guidelines [28]. Growth controls consisting of medium and medium with ethanol were included. Inoculated 96-well microtiter plates were covered with adhesive seals and incubated at 30°C for 4/5 days. Bacterial growth was controlled by visual inspection. The minimum bactericidal concentration (MBC) of CAR was determined by sub-culturing 10 μL of each microdilution on MHA plates followed by incubation at 30°C for 4–5 days. All experiments were performed in triplicate.

### Vapour contact assay

The effect of CAR vapours was evaluated with an invert Petri dishes method as previously described [31,32]. Briefly, CAMHA was inoculated with 5 μL of a suspension of the strain (10^4^ CFU/mL). Different volumes of CAR were added in a glass slide placed in the upper lid of each Petri dish. The final concentration ranging from 4096 to 8 μg/mL of the air space. The plates were sealed with adhesive seals and incubated at 30°C for 4–5 days. Each assay was made in triplicate. The lowest concentration of CAR vapours preventing visible growth was recorded as MIC.

### Effect of CAR on biofilm

The biofilm-forming ability of *Mycobacterium* strains was tested on 96-well polystyrene flat-bottomed microtitre plates as previously described [19]. Then, the effect of exposure of biofilm to CAR was determined either on biofilm formation and preformed biofilm.

#### Effect on biofilm formation

The effect of sub-inhibitory concentrations (sub-MICs) of CAR (ranging from 1/2 × MIC to 1/16 × MIC) on biofilm-forming ability was studied [33]. Bacterial cultures, were grown for 3–4 days in MBB + 0.5% glycerol + 10% OADC, standardized to 1 × 10^8^ CFU/mL by optical density (OD_492_) measurements and diluted 1:100 was inoculated (100 μL) into each well of microtiter plates in the presence of sub-MICs of CAR (100 μL) or medium (control). The correlation between CFU/mL and optical density (OD_492_) was obtained through the use of a standard curve and the following equation which was developed by plotting the OD values as a function of the log CFU/mL:

y=2E−10x+0.0296;R2=0.9683 (1)

After incubation for 3–4 days at 37°C, the effect on planktonic bacterial growth i) and biofilm biomass ii) was estimated as follows:

- the medium (planktonic bacterial growth) was removed from the each well and transferred to wells of a new 96-well polystyrene flat-bottomed microtitre plates in order to evaluate the total mass amount by measuring the OD_492_ using a spectrophotometer EIA reader (Bio-Rad Model 2550, Richmond, CA, USA);
- the biofilm biomass was evaluated as follows: the wells of microtitre plates were washed twice with sterile PBS, dried, stained with 0.1% safranin and washed with water. The biofilm biomass eluted in acetic acid 30% (v/v) was evaluated by OD_492_ measurements. The biofilm reduction was estimated by the following equation:

$$\left[100-(\frac{{OD}_{492\;of\;CAR\;well}}{mean\;{OD}_{492\;of\;control\;well}})\right]\;x\;100$$ (2)

All experiments were made in triplicate.

#### Effect on preformed 4 and 8 day-biofilms

Strains grown for 3–4 days in MBB + 0.5% glycerol + 10% OADC were standardized to 1 × 10^6^ CFU/mL and were inoculated (200 μL) in 96-well polystyrene flat-bottomed microtitre plates. After incubation for 4 and 8 days at 37°C, the planktonic bacterial growth was dislodged and the wells were washed with sterile PBS and filled with twofold dilutions of CAR, ranging from the MIC to a 16-fold the MIC (referred to the values reported in Table 2). After incubation for 4 days at 37°C, the effects on i) biofilm supenatant growth ii) biofilm biomass and iii) biofilm metabolic activity were estimated as follows.

- Biofilm supernatant growth. To determine whether CAR treatment prevented the growth in the biofilm supernatant, the medium was removed from the each well and transferred to wells of a new 96-well polystyrene flat-bottomed microtitre plates in order to evaluate the total mass amount by measuring the OD_492_;
- Biofilm biomass. The planktonic growth was dislodged and each well was washed with PBS, dried, stained with 0.1% safranin and then washed with water. The biofilm biomass was eluted in acetic acid 30% (v/v) and the OD_492_ was quantified.
- Biofilm metabolic activity. The planktonic growth was dislodged and, after washing, each well was treated with the Cell Proliferation Kit II XTT (Roche Diagnosis, Mannheim, Germany) as previously reported [34]. This assay is based on the metabolic reduction of a tetrazolium salt [2,3-bis[methyloxy-4-nitro-5-sulfophenyl]-2H-tetrazolium-5carboxanilide carboxanilide (XTT)] to a colored water-soluble formazan derivative. Briefly, each well was filled with the XTT solution (final concentration 0.3 mg/mL) for 5 h at 37°C and the formazan derivative was measured spectrophotometrically at 492 nm.

**Table 2:** Antibacterial activity of CAR, in liquid and vapour phase, against RGM strains.

Strain #	*Mycobacterium* spp.	MIC BM^a^	MBC BM^a^	MIC VC^b^
09716	*M*. *abscessus*	128	256	64
29904	*M*. *abscessus*	256	512	16
30235	*M*. *abscessus*	512	2048	16
70513	*M*. *abscessus*	64	128	16
73596	*M*. *abscessus*	64	128	16
90459	*M*. *abscessus*	64	128	16
74600	*M*. *abscessus*	64	128	16
74471	*M*. *chelonae*	64	128	16
26647	*M*. *fortuitum*	64	256	16
45646	*M*. *mucogenicum*	64	64	16
44041	*M*. *smegmatis*	32	128	16

^**a**^ BM, broth microdilution method: values are given as μg/mL.

^**b**^VC, vapour contact method: values are given as μg/mL air space.

All experiments were made in triplicate.

The reduction of biofilm supernatant growth/biofilm biomass/biofilm metabolic activity was estimated using Eq 1. The action of CAR was classified according to Lemos et al. [35] classification, as follows: < 25%—low efficacy; 60%—moderate efficacy; 60 ≤—< 90%—high efficacy; 90 ≤—≤ 100%—excellent efficacy.

### Statistical analysis

ANOVA was used to evaluate the significant differences between the samples treated with CAR and the samples without CAR. A *p* value <0.05 was assumed as significant.

## Results

### Susceptibility of RGM strains to antibiotics and CAR

The results of susceptibility tests showed that *M*. *abscessus* strains were resistant to AMK (#90459, MIC 64 μg/mL), CIP (#09716, #29904, #30235, #70513, #73596, #90459, #74600, MIC ≥ 4 μg/mL), CLR after 72 h incubation (#29904, #70513, #73596, MIC 8 μg/mL), CLR after 14 incubation days (#09716, #29904, #30235, #70513, #73596, #74600, MIC > 8 μg/mL), LZD (#09716, #29904, #70513, #73596, #90459, MIC 32 μg/mL), MEM (#29904, #73596, #74600, MIC > 16 μg/mL and #09716, #70513, MIC 16 μg/mL), SX (#09716, #29904, #70513, #73596, #90459, #74600, MIC > 64 μg/mL), TGC (#29904, MIC 8 μg/mL) and intermediate to AMK (#09716, #29904, #70513, #73596, #74600, MIC 32 μg/mL), FOX (#29904, #70513, #73596, MIC 32 μg/mL), LZD (#74600, MIC 16 μg/mL); *M*. *chelonae* #74471 was resistant to AMK (MIC 64 μg/mL), FOX (MIC > 128 μg/mL), CIP (MIC > 4 μg/mL), MEM (MIC >16 μg/mL), SX (MIC > 64 μg/mL) and intermediate to LZD (MIC 16 μg/mL); *M*. *fortuitum* #26647 was resistant to CIP (MIC 4 μg/mL), CLR after 14 incubation days (MIC > 8 μg/mL), SX (MIC: > 64 μg/mL) and intermediate to CLR after 72 h of incubation (MIC 4 μg/mL), FOX (MIC 32 μg/mL); *M*. *mucogenicum* #45646 was resistant to CIP (MIC > 4 μg/mL), SX (MIC > 64 μg/mL); *M*. *smegmatis* #44041 was resistant to SX (MIC > 64 μg/mL).

The susceptibility patterns of RGM strains to CAR, in liquid and vapour phase are shown in Table 2. The MIC values obtained using microdilution technique were equal to 64 μg/mL for 7 out of 11 RGM isolates; the MICs of the remaining four *M*. *abscessus* isolates were 32 μg/mL (#44041), 128 μg/mL (#09716), 256 μg/mL (#29904) and 512 μg/mL (#30235). The MBC values were 2–4 times higher than MICs (from 64 μg/mL to 2048 μg/mL) in all RGM strains, except for one isolate (# 45646) for which the MIC was equal to the MBC value. The MIC values obtained using vapour contact assays were lower than those in liquid medium for all the RGM isolates. Specifically, MICs were equal to 16 μg/mL for all strains except for one of them (#09716), for which the MIC was 64 μg/mL.

### Efficacy of CAR on biofilm formation

Biofilm biomass measurements showed the efficacy of CAR against biofilm formation of randomly selected *Mycobacterium* strains, i.e. *M*. *abscessus* (n = 4;) and *M*. *fortuitum* (Table 3). Although concentrations of 1/2 × MIC and 1/4 × MIC produced a relative inhibition of planktonic growth (28–46%), these concentrations showed a strong inhibition of biofilm (61–77%) in respect to 1/8 × MIC and 1/16 × MIC (Table 3); notably, CAR at 1/2 × MIC caused reductions of biofilm biomass accumulation ≥ 70% for all tested strains. According to Lemos et al. [35] classification, CAR at 1/2 × MIC and 1/4 × MIC was classified as highly effective (60% ≤ inhibition value < 90%).

**Table 3:** Effect of CAR at sub-MICs on planktonic growth and biofilm formation.

Strain	CAR sub-MIC	Planktonic Growth		Biofilm Biomass	
		OD_492_	Reduction (%)	OD_492_	Reduction (%)
*M*. *abscessus* #09716	1/2	0.382±0.022	35	0.365±0.010	71
	1/4	0.410±0.012	31	0.464±0.018	63
	1/8	0.432±0.025	27	0.715±0.027	43
	1/16	0.482±0.031	18	0.863±0.045	31
	Control	0.590±0.034		1.256±0.038	
*M*. *abscessus* #29904	1/2	0.343±0.031	40	0.351±0.041	70
	1/4	0.410±0.022	28	0.440±0.030	63
	1/8	0.450±0.041	21	0.720±0.052	39
	1/16	0.482±0.012	15	0.854±0.043	28
	Control	0.568±0.033		1.180±0.180	
*M*. *abscessus* #70513	1/2	0.358±0.009	47	0.312±0.008	72
	1/4	0.425±0.011	38	0.425±0.012	61
	1/8	0.532±0.015	22	0.625±0.024	42
	1/16	0.653±0.024	4	0.958±0.028	11
	Control	0.682±0.022		1.083±0.026	
*M*. *abscessus* #73596	1/2	0.343±0.012	45	0.384±0.010	73
	1/4	0.405±0.014	35	0.425±0.025	70
	1/8	0.442±0.024	29	0.742±0.042	48
	1/16	0.486±0.033	22	0.825±0.038	42
	Control	0.625±0.041		1.425±0.034	
*M*. *fortuitum* #26647	1/2	0.405±0.025	46	0.372±0.032	77
	1/4	0.532±0.022	29	0.427±0.041	73
	1/8	0.736±0.033	2	0.537±0.028	66
	1/16	0.743±0.028	1	0.693±0.052	56
	Control	0.752±0.052		1.592±0.043	

Data are presented as the mean ± standard deviation of three independent experiments.

### Efficacy of CAR on preformed 4 and 8 day-biofilms

**Fig 1:**
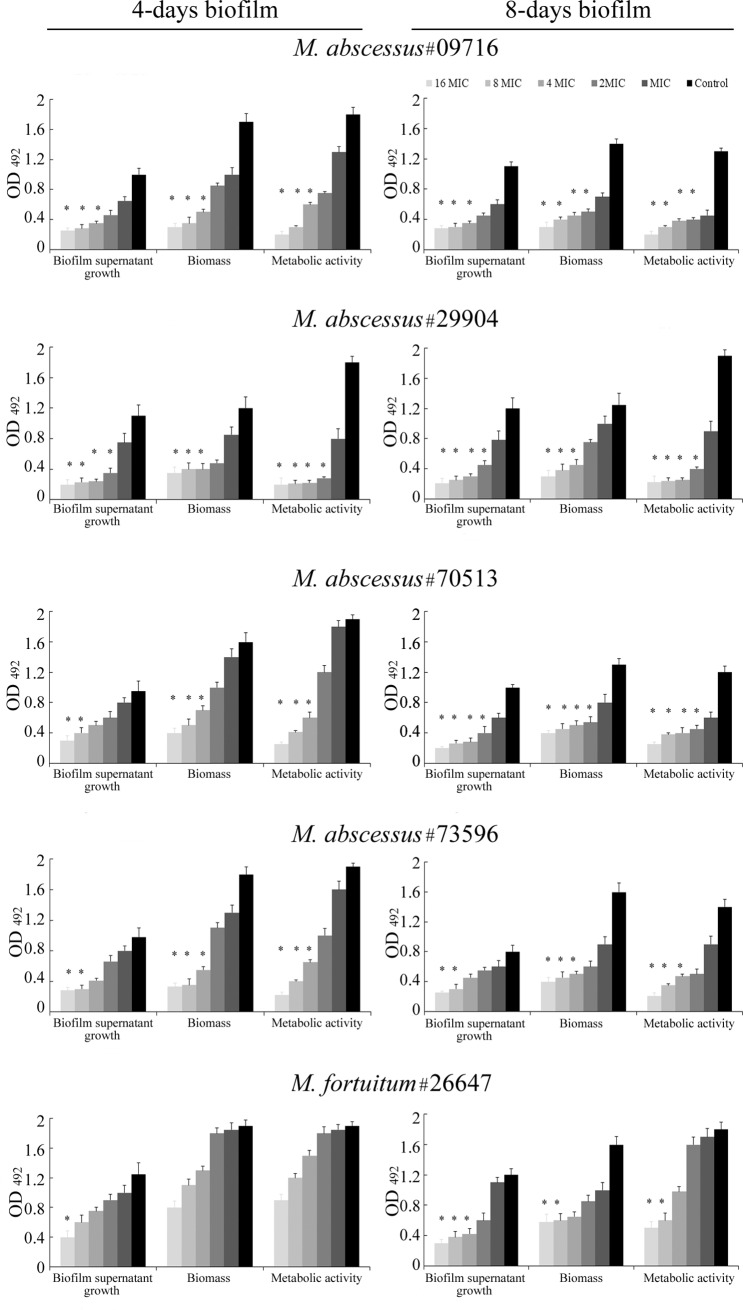
Activity of CAR against 4- and 8-days old rapidly growing mycobacteria biofilms. Effects of different concentrations, ranging from MIC to 16 x MIC, on biofilm supernatant growth, biofilm biomass and biofilm metabolic activity. * indicate significant differences between the samples treated with CAR and the samples without CAR (ANOVA, *p <* 0.05).

The effect of CAR on preformed biofilms at two different maturation stages (4- and 8-days old) was evaluated in terms of influence on biofilm supernatant growth, biofilm biomass and biofilm metabolic activity (Fig 1). CAR showed a significant high efficacy (60 ≤–< 90%) against preformed biofilm at concentrations slightly higher respect to those on the planktonic phase (from 2 × MIC to 8 × MIC). Furthermore, 4 d- and 8 d-preformed biofilm showed a similar susceptibility profile for *M*. *abscessus* #29904 and *M*. *abscessus* #73596 whereas showed a susceptibility related to the maturation stage for *M*. *abscessus* #09716 and *M*. *abscessus* #70513. On the contrary, for *M*. *fortuitum* a minor effect of CAR on both 4 and 8 days old biofilms was observed.

Specifically for *M*. *abscessus* #29904, the effective percentage reductions of biofilm supernatant growth (68% - 62%, for 4 d- and 8 d-biofilm), biofilm biomass (67% - 64%, for 4 d- and 8 d-biofilm) and biofilm metabolic activity (84% - 79%, for 4 d- and 8 d-biofilm) were detected with 2 × MIC, 4 × MIC and 2 × MIC, respectively. Moreover, even using increasing CAR concentrations (from 2 x MIC to 16 x MIC), similar OD results for 4 d-biofilm were detected. This evidence is probably related to the higher MIC value of CAR (256 μg/&mL) respect to most RGM (64 μg/mL).

For *M*. *abscessus* #73596, the effective percentage reductions of biofilm supernatant growth (69% - 62%, for 4- and 8 d-biofilm), biofilm biomass (70% - 69%, for 4- and 8 d-biofilm) and biofilm metabolic activity (66%, for 4- and 8 d-biofilm) were detected with 8 × MIC, 4 × MIC and 4 × MIC, respectively.

Differently, a more susceptibility of 8 d- respect to 4 days old biofilm was detected for *M*. *abscessus* #09716 and *M*. *abscessus* #70513. Despite effective percentage reductions (65% - 68%, for 4 d- and 8 d-biofilm) of biofilm supernatant growth of *M*. *abscessus* #09716 were achieved with the same concentration of CAR (4 × MIC), the significant biofilm biomass and biofilm metabolic activity decrease (> 65%) was detected with 4 × MIC for 4 d-biofilm and 2 × MIC for 8 d-biofilm. Equally, significant reduction of biofilm supernatant growth (> 60%) of 4 d- and 8 d-biofilm *M*. *abscessus* #70513 was achieved with 4–8 × MIC and 2 × MIC, respectively.

## Discussion

The search of novel anti-mycobacterial agents for the control of biofilm growth mode is crucial. At this regard, we have recently reported the antimicrobial activity of curcumin, a phenolic compound extracted from the *Curcuma longa*, against a lung isolate of *M*. *abscessus* [33]. CAR has been identified as a novel small molecule with activity against *M*. *avium* subsp. *paratuberculosis* and as a monoterpenic inhibitor of the chorismate mutase enzyme of *M*. *tuberculosis* [36, 37]. Moreover, CAR showed to be selective for *M*. *tuberculosis*, have efflux pumps activity and induce rough bacillary and agglomerates [38]. However, few data demonstrated the efficacy of CAR against RGM [26]. The present study demonstrated a good activity of CAR against resistant RGM isolates with MICs equal to 64 μg/mL for most strains and MBC values from 2 to 4 times higher. This suggests interesting applicative prospects as new anti-RGM agent. CAR could be considered a valuable support in the therapeutic treatment of RGM also for topical treatment of mucous, skin and wounds microbial infections [39]. In addition, CAR is a promising molecule with a potential in disinfection pratice e.g. in drinking water distribution systems. Furthermore, the activity of CAR vapours against RGM strains were first shown: CAR in vapour phase has shown higher antimicrobial activity (MICs equal to 16 μg/mL for most strains) than that revealed in liquid state. Some evidences proved that the effect of vapours is due to the combined action of deposition of CAR on bacteria together with adsorption through the agar medium [40,41]. Carvacrol is a volatile molecule and the effectiveness of its vapours without requiring direct contact could have additional applicative advantages. In this context, the administration of carvacrol by inhalation could implement the strategies in the treatment of RGM. With regard to the development of new preparations for inhalation therapy, Houdkova et al. [42] described the antibacterial potential of CAR in the liquid and vapour phase and its low cytotoxicity in lung fibroblast cells MRC-5. The easy penetration of vapours into inaccessible areas could be exploited for the treatment of extrapulmonary RGM diseases such as otomastoiditis or chronic otitis media [43,44]. Specifically, the potential role of CAR in the inhalation therapy or in the treatment of acute otitis media against *Hemophilus infuenzae*, *Streptococcus pyogenes*, *Streptococcus pneumoniae*, and *Staphylococcus aureus* pathogens [40,45] has been studied. Finally, a further use of CAR vapours by airing could concern the environmental remediation in order to reduce the persistence of RGM, e.g. in hospital sinks, showerheads and homes and therefore the risk of infection.

Rapidly growing mycobacteria have been studied for their ability to form biofilm [9]. CAR emerges as promising antimicrobial molecule with high potential for the control of microorganisms that are currently difficult to treat either as free- and sessile-growth lifestyle organisms [12,17–19,40,46,47]. In this context, the obtained results on the biofilm taking into account the limit of the study related to the use of only one species of *M*. *fortuitum* represent another important finding. Concentrations below the MIC (1/2–1/4 × MIC) impaired biofilm formation whereas concentration above the MIC (2–8 × MIC) caused significant disaggregation effect on the biofilm biomass and metabolic viability of cells embedded in a biofilm matrix at two maturation state (4-day and 8-day biofilms). According to Lemos et al. [35] classification, this action of CAR was classified as highly effective because the percentage of reduction ranged from 60% to 90% (60 ≤—< 90%). Regarding the influence of biofilm maturity on CAR activity, we observed that the 8-day biofilm of *M*. *abscessus* was more sensitive than the 4-day biofim. The effect could be due to a poorer 8-day biofilm in terms of biomass and metabolic activity. In contrast, the 4- and 8-day biofilm of *M*. *fortuitum* showed a similar susceptibility trend. About CAR toxicity, Cacciatore et al. [48] demonstrated that the toxic concentration of CAR (hemolytic activity on human red cells) was more than 6200 μg/mL. In the light of this, except for the highest value of 16 x MIC, corresponding to 8192 μg/mL when MIC value was equal to 512 μg/mL, the other concentrations were under 6200 μg/mL.

## Conclusion

In conclusion, within the limits of the present study CAR could have the potential for implementation of strategies for treating *Mycobacterium* strains also in a sessile lifestyle and offers interesting applicative prospects related to its volatility such as diffusion and penetration into inaccessible areas.
